# An Apology for the East Wind

**Published:** 1888-03-03

**Authors:** 


### The Doctor's Pulpit.

### AN APOLOGY FOR THE EAST WIND.

Nero has been "whitewashed," and Henry the Eighth proved the best of husbands. Why, then, should no plea be entered on behalf of the East Wind? This age is nothing if not critical. It prides itself on looking at all things dispassionately and with the coldest impartiality. So be it! Let the East Wind, as well as other monsters, have its meed of justice.

We will not begin by denying the vices and even terrors of our client. Let it be granted that the name of Richard Cœur de Lion was not a more dread sound to an infant Syrian than is the mention of an east wind to many a shivering southerner. Let it also be freely admitted that the danger is not fancied but most real, and is founded on the sure and certain ground of personal experience. Let us go even further, and proclaim aloud that February, March, April, and often May, are amongst the most dangerous months of the year, and that of all the enemies which stalk abroad in these changing periods, by far the most cruel is the east wind. Nay, let the most flagrant outrages upon man and beast which this dread wind produces—including, as they do, pneumonia, pleurisy, bronchitis, asthma, neuralgia, sciatica, disordered liver, ruined digestion, maddened temper, black melancholy, and a host of other diseases which would make even a Mark Tapley confess himself defeated—let all these horrid spectres be dragged into open court and exhibited before judge, jury, and spectators—it is still possible to say at least one good word on behalf of the prisoner at the bar.

The advocate, it may be explained, does not speak without knowledge—the knowledge bought in the costly market of experience. He thinks he has learnt the art of dealing with his foe, and even of turning him into a friend.

The gist of our defence of an almost universal enemy may be expressed in the words of that proverb of the vulgar, "What is one man's meat is another man's poison." To some men, and those not a few, the east wind acts as a fine bracing, invigorating tonic. They go out in the early morning, fortified with a good breakfast of ham and eggs and hot coffee, and in five minutes they walk themselves into a cheerful glow and the briskest of pedestrian moods. Whereas on a dull, foggy day they are tired at the end of three or four miles, on a dry day such as the east wind produces they can walk ten or twelve without a sign of fatigue. Precisely this tonic effect is produced upon domestic animals. Your Scotch terrier, on a cold, clear morning, rushes about with tail in air and excited bark, as if he had breakfasted off champagne and oysters. Your horse stands chafing and dancing, hardly waiting for you to get firm in the saddle before he is off at full trot, or even at a sharp gallop, if you permit him. Those most staid and dignified of all civilised quadrupeds, the dairy cows, toss their heads, fling aloft their tails, and career at breakneck speed for their morning drink at the brook, instead of marching, as they usually do, in the slowest of processions, like hired mourners at a funeral.

It is this quality of dry crispness which gives its tonic and stimulating characters to an east wind. And there seem to be excellent physiological reasons why these effects should be produced. Any one accustomed to observe himself and his own moods and tenses knows that his hands are smaller on a dry east-windy day than on a moist warm one. A look at those portions of his body which are exposed will show him that his skin is shrunk and leathery-looking, instead of being in its ordinarily plump and distended condition. Why is this? The cold, dry air has contracted the surface blood-vessels and greatly diminished their contents. But the blood is still in the body, and being driven from the skin by the cold has fled to the deeper and warmer parts. Hence, not merely the heart, lungs, liver, and kidneys have received a larger supply, but the brain and spinal cord have also shared in the new wealth. The brain and cord being thus more richly supplied with material, a rapid rise of spirits, an increase of muscular energy, and an added power of endurance are promptly produced.

But there is another and possibly still more efficient cause of high spirits and power of work in the east wind. It causes a rarefaction of the air; and clear, rarefied air oxygenates the blood more quickly and effectively than heavy, stagnant air, and thus quickens the speed of every physiological process, hurrying on the whole life, as it were, at express instead of parliamentary speed. It produces all the exhilarating effects of the most powerful kind of stimulant without any of the objectionable consequences.

The statements now made need not be disputed. If any reader is sceptical let him try a twelvemonth in Edinburgh where the east wind is known at its direst. He will find that not only are the inhabitants of the city in the briskest of spirits at spring time, but that he himself has his full share of the pleasures of exuberant health. Though many persons are ignorant of the experiences here set forth, many others are perfectly familiar with them. This general affirmation may therefore be held as established—that to many people the east wind is a bracing, stimulating tonic, and has the same effect as a visit to the seaside. Those unfortunates who have never tasted the atmospheric champagne of March will probably ask, "Is it possible for us also to act the part of successful evangelists and to convert our enemy into a friend?" It is possible for some to bring about this much-to-be-desired result, but it can only be, as it so often is in moral affairs, by a preliminary process of self-conversion. There must first be acquired a sound digestion, habits of daily energetic and sufficiently prolonged exercise in the open air, and a fairly high level of general health. To this must be added the wearing of stout woollen garments from head to foot, with additional folds, if need be, round the loins and on the chest. The throat in many cases needs to be protected from the keen air by a silk (not a woollen) muffler, and the hands by warmly-lined gloves. Regular, sufficient, and suitable meals—including as much fat, in the shape of meat fat, butter, or cod liver oil as can be digested—are indispensable. Thus furnished within and without, a very great many people who now dread the east wind, might face it, not only without dismay, but with positive pleasure and a sense of triumphant victory.

But there are some who never have conquered this dread enemy, and who can never by any possibility hope to do so. For such the only course is to avoid it as much as possible by remaining indoors; keeping the dining, bedroom, and office fires in full activity; clothing themselves with the warmest of furs by day and in the softest of flannel gowns and eider downs by night. If, as will frequently happen, they must perforce go out of doors, they should never face the weather when hungry, never leave behind their overcoats, gloves, or mufflers, never sit in rooms without fires, never forget that in every breath of air an enemy lurks—an enemy who may drive them to desperation with a congested liver, torture them with the agonies of sciatica, or kill them in three days with pleuro-pneumonia. The east wind—like fire, water, or a shrewish wife—may be a good servant, but is a very bad master.

